# Acquisition of *glial cells missing 2* Enhancers Contributes to a Diversity of Ionocytes in Zebrafish

**DOI:** 10.1371/journal.pone.0023746

**Published:** 2011-08-17

**Authors:** Takanori Shono, Daisuke Kurokawa, Tsutomu Miyake, Masataka Okabe

**Affiliations:** 1 Department of Anatomy, The Jikei University School of Medicine, Tokyo, Japan; 2 Misaki Marine Biological Station, The University of Tokyo, Kanagawa, Japan; Ecole Normale Supérieure de Lyon, France

## Abstract

*Glial cells missing 2* (*gcm2*) encoding a GCM-motif transcription factor is expressed in the parathyroid in amniotes. In contrast, *gcm2* is expressed in pharyngeal pouches (a homologous site of the parathyroid), gills, and H^+^-ATPase–rich cells (HRCs), a subset of ionocytes on the skin surface of the teleost fish zebrafish. Ionocytes are specialized cells that are involved in osmotic homeostasis in aquatic vertebrates. Here, we showed that *gcm2* is essential for the development of HRCs and Na^+^-Cl^−^ co-transporter–rich cells (NCCCs), another subset of ionocytes in zebrafish. We also identified *gcm2* enhancer regions that control *gcm2* expression in ionocytes of zebrafish. Comparisons of the *gcm2* locus with its neighboring regions revealed no conserved elements between zebrafish and tetrapods. Furthermore, We observed *gcm2* expression patterns in embryos of the teleost fishes Medaka (*Oryzias latipes*) and fugu (*Fugu niphobles*), the extant primitive ray-finned fishes *Polypterus* (*Polypterus senegalus*) and sturgeon (a hybrid of *Huso huso* × *Acipenser ruhenus*), and the amphibian *Xenopus* (*Xenopus laevis*). Although *gcm2*-expressing cells were observed on the skin surface of Medaka and fugu, they were not found in *Polypterus*, sturgeon, or *Xenopus*. Our results suggest that an acquisition of enhancers for the expression of *gcm2* contributes to a diversity of ionocytes in zebrafish during evolution.

## Introduction

Developmental evolutionary studies have played an important role in elucidating the molecular mechanisms that give rise to morphological divergence and acquisition of new morphological structures and/or functions. These studies have proposed a model for the evolution of a novel morphological structure by modification of temporal and spatial patterns of gene expression; that is, changes in gene regulatory networks by adaptive mutations of transcription factors, including *cis*-regulatory elements [1]–[3]. However, only a few actual examples have been reported. In our current study, we focused on the *glial cells missing* (*gcm*) family and investigated the evolution of ion-regulating organs in vertebrates that may have evolved by changing the molecular developmental mechanisms of gene regulation of the *gcm* family.

*gcm* encodes a GCM-motif transcription factor in *Drosophila* and functions as a determinant in glia/neuron and plasmatocyte/macrophage cell fates [4]–[9]. Homologues of *Drosophila gcm*, *gcm1* and *gcm2,* have been isolated from vertebrates [10]–[12], and both genes are expressed in non-neuronal organs with diverse functions [12]–[20]. Mouse *gcm1* is expressed in the thymus, kidney, and placenta, where it is required for chorio-allantois morphogenesis [17], [20]. In contrast, mouse *gcm2* is expressed in the third pharyngeal pouch and the parathyroid gland that develops from the third pouch and is an essential gene for their development [21].

*gcm2* is expressed in pharyngeal pouches of the teleost fish zebrafish and the small-spotted catshark *Scyliorhinus canicula*. Its expression patterns suggest that the evolutionary origin of the parathyroid organ may be traced back to the pharyngeal pouches of fishes [22]–[24]. Furthermore, it has been reported that *gcm2* is expressed in H^+^-ATPase–rich cells (HRCs), a subset of ionocytes that are rich in H^+^-ATPase and located in the skin of zebrafish [25], [26]. Ionocytes are ion transporters with abundant mitochondria and extensive basolateral membrane infoldings that form a tubular system [27]. They are located in gills and scattered over the skin surface of aquatic vertebrates but are not found in amniotes. They play a central role in maintaining body fluid homeostasis by actively taking up and excreting ions [28]–[30].

In our current study, we showed that *gcm2* is essential for the development of HRCs and Na^+^-Cl^−^ co-transporter–rich cells (NCCCs), another subset of ionocytes in the skin of zebrafish. We also identified *gcm2* enhancers specific for the skin of zebrafish that are upstream and downstream of the *gcm2* locus. These enhancers were not found in the syntenic regions of tetrapods. Furthermore, we investigated *gcm2* expression patterns in embryonic stages of the teleost fishes Medaka (*Oryzias latipes*) and fugu (*Fugu niphobles*), the primitive ray-finned fishes *Polypterus* (*Polypterus senegalus*) and sturgeon (a hybrid of *Huso huso* × *Acipenser ruhenus*), and the amphibian *Xenopus* (*Xenopus laevis*). *gcm2*-expressing cells were observed in the skin of Medaka and fugu but not in *Polypterus*, sturgeon, or *Xenopus* embryos. We will discuss an importance of our finding of *cis*-elements for ionocytes in the skin of zebrafish.

## Results

### Zebrafish *gcm2* is essential for morphogenesis of ionocytes on the skin surface

**Figure 1:**
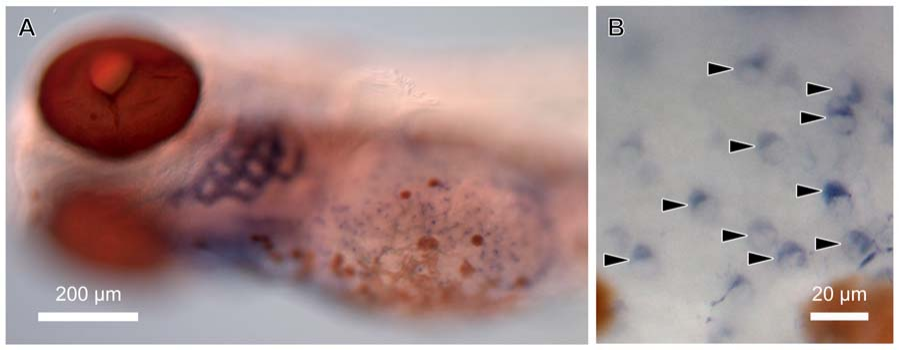
Expression of *gcm2* in the gills and the cells on the skin surface of zebrafish. Whole-mount *in situ* hybridization of 5-day-old zebrafish with *gcm2* probes. (A) *gcm2* is expressed in the gills. (B) Magnification of a yolk sac area. Some cells in the yolk sac express *gcm2* (arrowheads).

**Figure 2:**
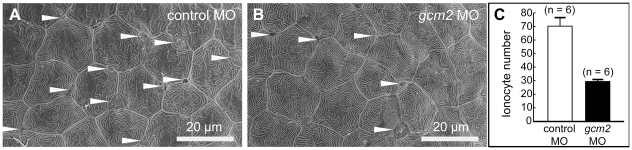
Scanning electron microscopy of the yolk sac membrane in 3-day-old zebrafish. (A, B) Magnification of the yolk sac membrane. (A) An embryo injected with a control morpholino (MO). (B) An embryo injected with a *gcm2* MO. (C) Quantitative comparison of the ionocyte number per 0.04 mm^2^ between embryos injected with control MO and *gcm2* MO. The ionocyte number on the yolk sac membrane area in *gcm2* morphants is less than that in control MO. Arrowheads indicate ionocytes. Because it is likely that cells were still alive or had differentiated without expression of a marker gene by loss of function, we observed the external morphology of the skin surface in morphants using scanning electron microscopy.

To confirm that *gcm2* is necessary for ionocyte development, zebrafish morphants injected with *gcm2* antisense morpholinos (MOs) were examined using scanning electron microscopy. The observations focused on yolk sac areas because expression of *gcm2* was localized in the gills and yolk sac (Figure 1A and B). In control embryos at 72 hours post-fertilization (hpf), ionocytes on the skin surface protruded between epithelial cells (Figure 2A), whereas in *gcm2* morphants at 72 hpf, the number of ionocytes was reduced. We observed a reduction of approximately 40% in the number of ionocytes on the skin surface per 0.04 mm^2^ when compared to the number of ionocytes in control embryos (Figure 2B and C). The reduction in the number of ionocytes in *gcm2* morphants suggested that *gcm2* MO treatment resulted in inhibition of ionocyte morphogenesis.

### Zebrafish *gcm2* is essential for the development of two of the three types of ionocytes

**Figure 3:**
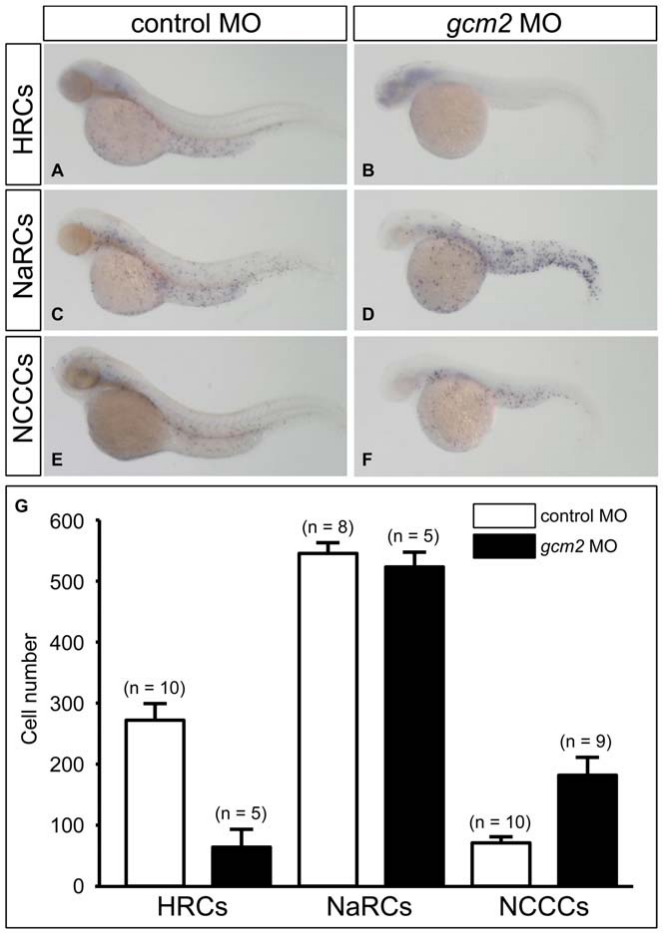
*gcm2* is essential for the development of HRCs and suppresses the development of NCCCs on the skin surface. (A, B) Whole-mount *in situ* hybridization of zebrafish 48 hours post-fertilization (hpf) with an *atp6v1al* probe as a HRC marker. (C, D) Whole-mount *in situ* hybridization with an *atp1b1b* probe as a NaRC marker. (E, F) Whole-mount *in situ* hybridization with a *slc12a10.2* probe as a NCCC marker. (A, C, E) Zebrafish (48 hpf) injected with a control morpholino (MO). (B, D, F) Zebrafish (48 hpf) injected with a *gcm2* MO. (G) Quantitative comparison of *atp6v1al*-positive cells, *atp1b1b*-positive cells, and *slc12a10.2*-positive cells in zebrafish injected with control MO and *gcm2* MO.

Although *gcm2* morphants showed a reduction in ionocyte numbers on the skin surface, it was not clear which types of ionocytes were reduced during morphogenesis. A recently proposed model suggests that there are at least three subtypes of ionocytes on the zebrafish skin surface: H^+^-ATPase–rich cells (HRCs); Na^+^-K^+^-ATPase–rich cells (NaRCs); and Na^+^-Cl^−^ co-transporter–rich cells (NCCCs) [28]. It has been reported that *gcm2* morphants are depleted of HRCs but not NaRCs [25], [26], although the status of NCCCs is unknown. To determine which types of ionocytes require *gcm2* for their development, we analyzed the expression patterns of ionocyte marker genes in *gcm2* morphants. In control embryos at 48 hpf, we detected with whole-mount *in situ* hybridization skin surface expression of *atp6v1al* (H^+^-ATPase subunit A) as a HRC marker gene [31], *atp1b1b* (Na^+^-K^+^-ATPase transporting beta 1b subunit) as a NaRC marker gene [31], and *slc12a10.2* (thiazide-sensitive Na^+^-Cl^−^ co-transporter, a member of the SLC12 family) as a NCCC marker gene [32] (Figure 3A, C, and E). In contrast, *gcm2* morphants showed a strong reduction in the number of HRCs, no significant change in the number of NaRCs, and an increase in the number of NCCCs (Figure 3B, D, F, and G). These results suggested that *gcm2* was essential for the development of HRCs, was not essential for the development of NaRCs, and suppressed the development of NCCCs.

### *zgcm2*-expressing cells on the skin surface are present only in teleosts

**Figure 4:**
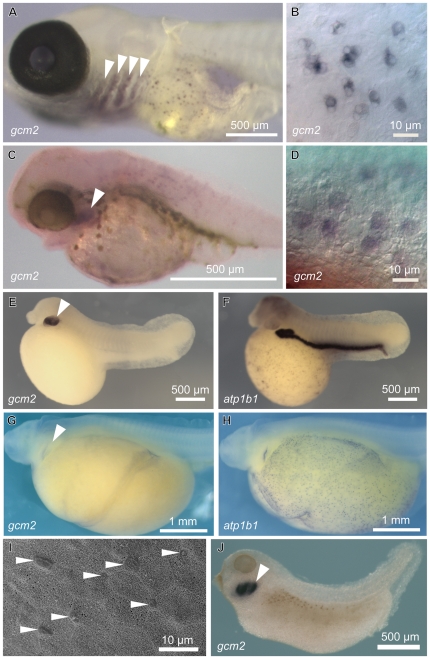
Expression of *gcm2* in Medaka, fugu, *Polypterus,* sturgeon and *Xenopus.* (A, B) Whole-mount *in situ* hybridization for *gcm2* in 13-day-old Medaka. (C, D) Whole-mount *in situ* hybridization for *gcm2* in 7-day-old fugu. (B, D) Magnification of the skin surface. *gcm2* is expressed in the gills (arrowheads) and the cells on the skin surface of the teleostean fishes, Medaka and fugu. (E, F) Whole-mount *in situ* hybridization for *gcm2* and *atp1b1* probes in 48-hpf *Polypterus*. (E) *gcm2* is expressed in the gills (arrowhead). (F) *atp1b1* is expressed in the kidney and the cells on the skin surface. (G, H) Whole-mount *in situ* hybridization for *gcm2* and *atp1b1* in sturgeon at stage 32. (G) *gcm2 is* expressed in the gills (arrowhead). (H) *atp1b1* is expressed in the cells on the skin surface. (I) Scanning electron microscopy on the yolk sac membrane of sturgeon at stage 32. Ionocytes (arrowheads) protrude between the epithelial cells. (J) Whole-mount *in situ* hybridization with the *gcm2* probe in *Xenopus* at stage 37. *gcm2* is expressed in the gills (arrowhead) but not on the skin surface of *Polypterus,* sturgeon, and *Xenopus* embryos.

To investigate if the induction of *gcm2* expression in ionocytes on the skin surface is unique to teleosts, we examined *gcm2* expression patterns in embryos of Medaka (13 days old), fugu (7 days old), *Polypterus* (48 hpf), sturgeon (stage 32), and *Xenopus* (stage 37). All embryos expressed *gcm2* in the pharyngeal pouches and gills (Figure 4A, C, E, G, and J). The Medaka and fugu embryos also expressed *gcm2* in a scattered pattern on the skin surface (Figure 4B and D). On the other hand, *Polypterus*, sturgeon, and *Xenopus* embryos did not express *gcm2* on the skin surface (Figure 4E, G, and J), although *Polypterus* and sturgeon embryos showed *atp1b1*-positive cells on the skin surface (Figure 4F and H). These results suggested that the cells expressing *gcm2* on the skin surface were unique to teleosts.

### Zebrafish 160 kb BAC includes *gcm2* enhancers specific for ionocytes on the skin surface

**Figure 5:**
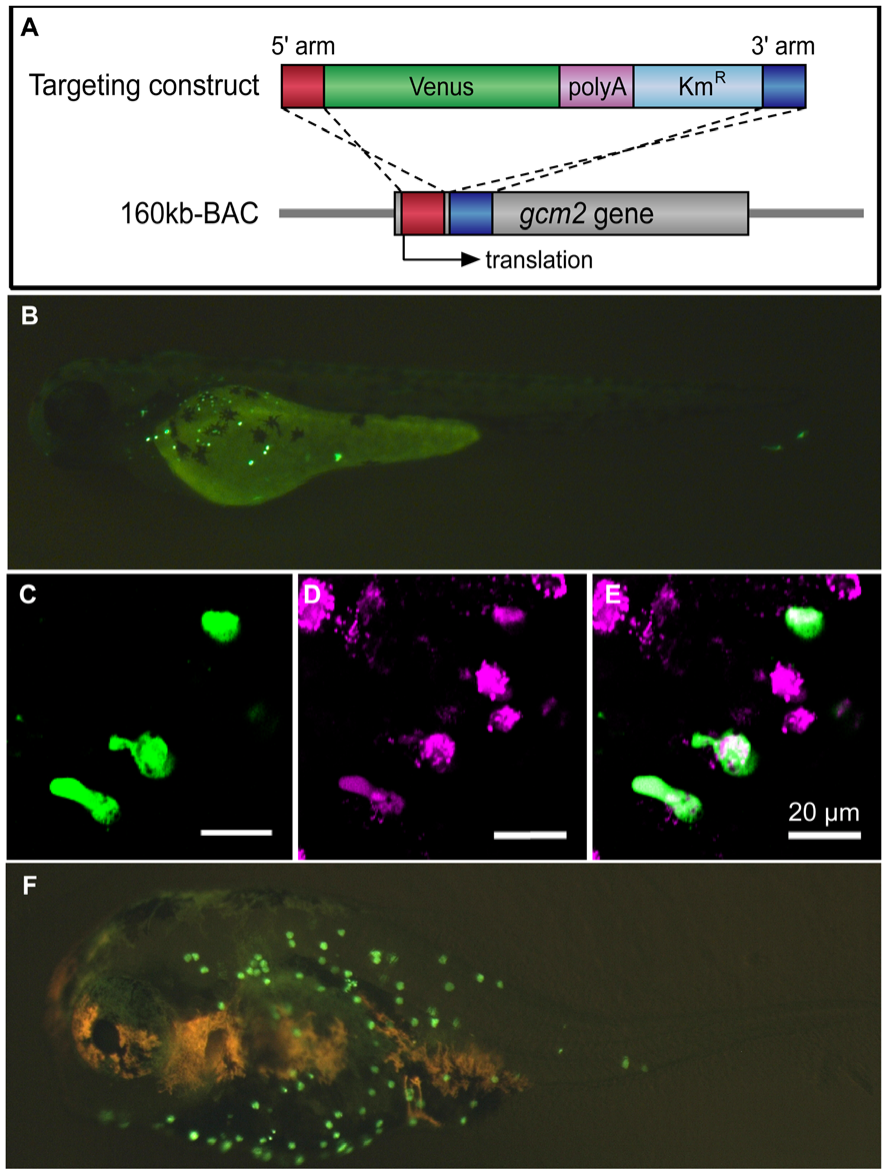
Analysis of *gcm2* enhancers specific for ionocytes on the skin surface of zebrafish. (A) Construction of the 160 kb BAC-Venus. The targeting construct was amplified by PCR from a plasmid containing Venus, polyA, and Km^r^. Each primer contained 50 bp of the *gcm2*-derived sequences that served as homology arms for homologous recombination. The 160 kb BAC contained sequences that were 120 kb upstream and 40 kb downstream of the *gcm2* locus. After homologous recombination, Venus was inserted into the translation site (160 kb BAC-Venus). (B) A 160 kb BAC-Venus transient transgenic zebrafish (72 hpf). Venus-expressing cells are observed on the skin surface but not in the gills. (C) Several cells on the yolk sac expressed Venus. (D) Ionocytes stained with MitoTracker. (E) Merge of (C) and (D), showing overlap in staining with a subset of ionocytes. (F) A 160 kb BAC-Venus transient transgenic 7-day-old fugu. Venus-expressing cells are observed on the skin surface.

Because *gcm2* was uniquely expressed in ionocytes on the skin surface of teleosts, the zebrafish was used to identify potential *cis*-regulatory elements (i.e., enhancers of *gcm2* that may regulate its expression on the skin surface). To examine whether putative enhancers are located near the *gcm2* locus, we prepared a 160 kb BAC (Bacterial Artificial Chromosome) clone containing zebrafish *gcm2* and its neighboring genes, *elongation of very long chain fatty acids like 2* (*elovl2*) and *male germ cell–associated kinase* (*mak*). Venus was then inserted into the *gcm2*-coding region of the 160 kb BAC clone, and this construct was injected into zebrafish eggs (Figure 5A). In transient transgenic embryos, Venus was detected on the skin surface, especially over the yolk sac membrane after 24 hpf, but it was not observed in the gills at any of the observed stages of development (Figure 5B). Because all Venus-expressing cells also were labeled with MitoTracker, an ionocyte marker that stains mitochondria (Figure 5C, D, and E), our results demonstrated that Venus-expressing cells in the transgenic embryos were ionocytes and that the 160 kb BAC-Venus clone contained *gcm2* enhancers specific for ionocytes in the skin. In addition, when the 160 kb BAC-Venus construct was injected into fugu embryos, Venus-expressing cells were observed on the skin surface (Figure 5F). This result suggested that there were common *trans* factors that induced *gcm2* on the skin surface in zebrafish, but whether the common factors are actually present in fugu remains to be investigated.

### *gcm2* enhancers are located 8 kb upstream and 41 kb downstream of *gcm2*

**Figure 6:**
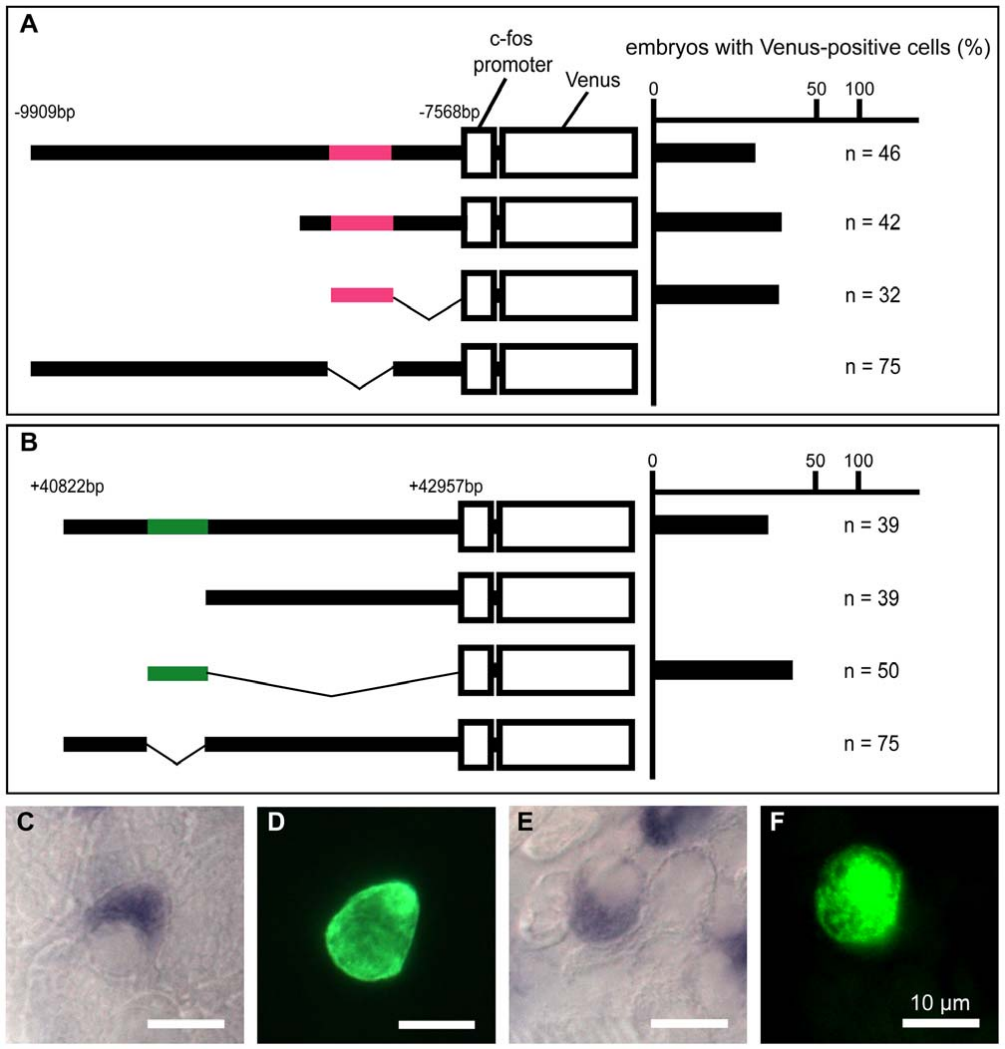
Transient transgenic analysis of the *gcm2* enhancer regions for HRCs specific to the skin surface. (A, B) The chart on the right shows the percentage of embryos with Venus-positive cells on the skin surface at 24 hpf. (C–F) *In situ* hybridization with an *atp6v1al* probe in combination with immunohistochemistry using anti-GFP on the yolk sac of transient transgenic 4-day-old zebrafish. (C, D) A zebrafish expressing pTolfV that contains the *gcm2* enhancer region that is 8 kb upstream. (E, F) A zebrafish expressing pTolfV that contains the *gcm2* enhancer region that is 41 kb downstream. The *gcm2* loci at −8249 to −8004 bp upstream and at +41277 to +41497 bp downstream were required for Venus expression in HRCs.

To characterize *gcm2* enhancers specific for ionocytes on the skin surface, several fragments derived from the zebrafish genomic region containing the *gcm2*, *elovl2*, and *mak* loci were PCR amplified, cloned into the *Tol2* transposon-based Venus expression vector (pTolfV), and injected into zebrafish eggs. Transient transgenic embryos injected with the pTolfV vector containing either the 8 kb upstream region or the 41 kb downstream region of the *gcm2* translation site showed Venus expression specifically in ionocytes on the skin surface (Figure 6A, B and Table S1). Moreover, all reporter constructs with the 8 kb upstream region between −8249 and −8004 bp (−8 kb enhancer) or the 41 kb downstream region between +41277 and +41497 bp (+41 kb enhancer) displayed Venus positivity, indicating that each region contained enhancers that specifically regulated expression of *gcm2* in ionocytes on the skin surface.

### Venus-expressing cells on the skin surface express a HRC marker gene

To examine whether Venus-expressing cells on the skin surface are HRCs, zebrafish embryos were injected with a construct containing the −8 kb or +41 kb enhancer and examined with *in situ* hybridization using an *atp6v1al* probe in combination with immunohistochemistry using anti-GFP. All Venus-expressing cells on the skin surface expressed *atp6v1al* mRNA (Figure 6C, D, E, and F), which demonstrated that the *gcm2* enhancers we identified were enhancers specific for HRCs in the skin.

### *gcm2* enhancer regions specific for HRCs in zebrafish are not found in tetrapods

**Figure 7:**
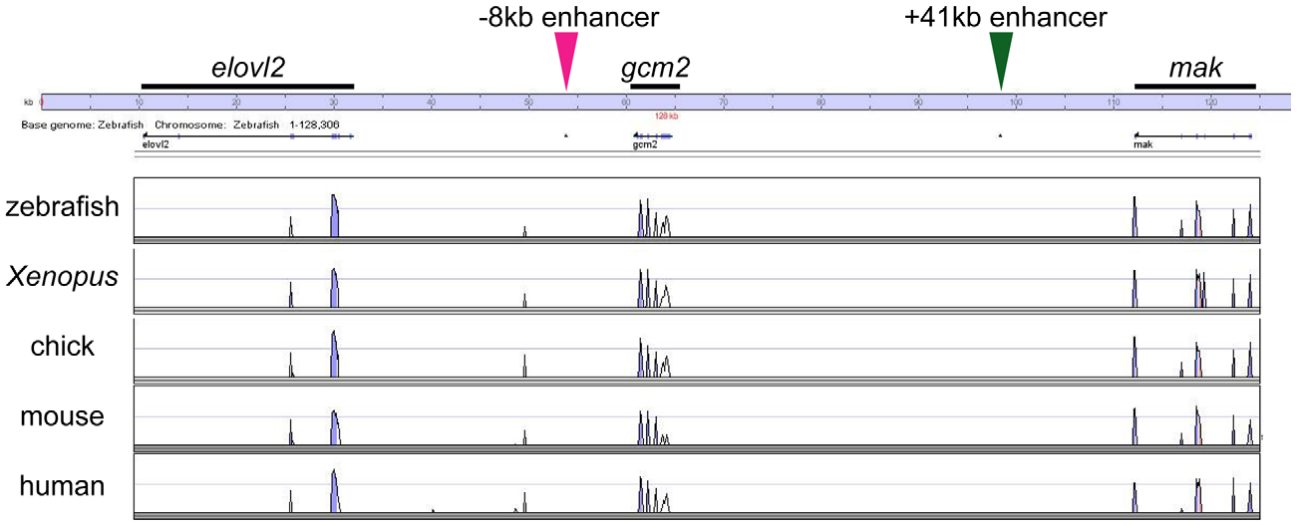
Two *gcm2* enhancers in zebrafish are not found in tetrapods. VISTA plots of zebrafish, *Xenopus*, chick, mouse, and human genomic sequences of the *gcm2* locus with its neighboring genes, *elovl2* and *mak*.

We used VISTA software [33] to examine whether the two *gcm2* enhancers are conserved among different species of vertebrates. Analysis of the *gcm2* locus and neighboring sequences in zebrafish and some tetrapods, including *Xenopus*, chick, mouse, and human, showed no conserved sequences in these vertebrate species (Figure 7).

## Discussion

The genes that regulate the specification of NaRCs, HRCs, and NCCCs from their common precursor cells in zebrafish remain to be determined. Previous studies [25], [26] and our current study suggest that regulation of *gcm2* plays a key role in the specification and differentiation of common precursor cells into each type of ionocyte. Ionocytes are derived from common precursor cells of keratinocytes [31], [34]. Furthermore, a positive regulatory loop involving *foxi3a* and *foxi3b* [31] regulates the specification and differentiation of HRCs and NaRCs; however, the genes that regulate the specification and differentiation of NCCCs remain unknown. Our current study using *gcm2* morphants showed that *gcm2* is involved in the differentiation of both HRCs and NCCCs on the skin surface. *gcm2* morphants showed not only extremely reduced numbers of HRCs but increased numbers of NCCCs (Figure 3). Therefore, we propose that *gcm2* functions as a binary switch in determining HRC or NCCC fate on the skin surface. Because *gcm* in *Drosophila* functions as a binary switch in determining glial or neuronal cell fate [4], [5], it is possible that vertebrate *gcm2* has a similar function.

If *gcm2* functions as a binary switch, then what *trans* factors directly regulate *gcm2* in progenitor cells of HRCs? *foxi3a* is a candidate gene because positive feedback of *gcm2* and *foxi3a* in the specification of HRCs may maintain the expression of *gcm2* [26]. This raises the question of whether *foxi3a* directly regulates *gcm2* enhancers. Because the −8 kb enhancer region contains a consensus sequence for a Fox protein binding site [35] (Figure S1), this binding site may directly control the −8 kb enhancer upon its binding to *foxi3a*. However, a transient transgenic zebrafish that was injected with a pTolfV vector containing the −8 kb enhancer region with a deletion of the Fox protein binding site did not lose Venus activation (Table S2). In addition, the +41 kb enhancer region did not contain a sequence identical to the Fox protein binding site (data not shown). These results and the genetic data of Esaki et al. (2009) suggest that *foxi3a* indirectly regulates *gcm2* enhancers [26].

It has been hypothesized that the acquisition of enhancers during the evolution of animals is an evolutionary opportunity for animals to obtain new cells and organs [1]–[3]. Because the enhancers we identified in our current study are required for the expression of *gcm2* in progenitor cells of HRCs, *gcm2* enhancers may have enabled zebrafish to acquire new HRC ionocytes. When the zebrafish 160 kb BAC-Venus construct that contained *gcm2* enhancers was injected into fugu embryos, Venus-expressing cells were observed on the skin surface (Figure 5F). Unfortunately, since the genome database of Medaka, fugu, and stickleback at the *gcm2* locus still contains unsolved sequences, it remains uncertain if *gcm2* enhancers of zebrafish are conserved in fugu and even other teleosts. Further studies will be needed to confirm our results.

Expression of *gcm2* on the skin surface of cells was identical among zebrafish, Medaka, and fugu (Figure 1 and 4); however, *gcm2* was not expressed in cells on the skin surface of the embryos of *Polypterus* or sturgeon, stem groups of actinopterygian fishes [36]–[38], or *Xenopus*, despite the fact that these animals have cells on the skin surface that are positive for ionocyte markers (Figure 4) [39], [40]. In addition, *gcm2* enhancer regions in zebrafish were not found in the synteny block that contained the *gcm2* locus and its neighboring regions in the tetrapods that we examined (Figure 7). All together, these results suggest that an acquisition of enhancers for the expression of *gcm2* contributes to a diversity of ionocytes in zebrafish. Whether the same enhancers are responsible for development of ionocytes in other teleosts including fugu remains to be investigated. Since *gcm2*-expressing cells were not observed in primitive actinopterygian fishes and because *gcm2* enhancers are not found in tetrapods, the evolution of *gcm2* enhancers is one of a few examples to provide a fish model for evolutionary developmental studies of a novel morphological structure through changes in gene regulatory networks after the teleost-specific whole genome duplication [41]–[43].

## Materials and Methods

### Animals

Zebrafish and Medaka were purchased at a local pet shop and maintained as described [44]. Fertilized eggs were incubated in E3 medium (5 mM NaCl, 0.17 mM KCl, 0.33 mM CaCl_2_, and 0.33 mM MgSO_4_) at 28.5°C. Fugu (*F. niphobles*) eggs were collected on Arai beach, Kanagawa prefecture, Japan, and incubated in artificial seawater, Tetra marine salt pro (Tetra), at 20°C. *Polypterus* (*P. senegalus*) was purchased at a local pet shop, and fertilized eggs were incubated in E3 medium. Sturgeon is a hybrid of *H. huso* × *A. ruhenus*. Their eggs were supplied by Fujikin Incorporated, and embryos were incubated in freshwater at 20°C. Embryos were staged according to Ballard and Needham (1964) [45]. *Xenopus laevis* was purchased at a *Xenopus* culture shop, and fertilized eggs were obtained *in vitro* using standard methods. Embryos were staged according to Nieuwkoop and Faber (1967) [46].

### Scanning electron microscopy

Zebrafish and sturgeon embryos were fixed in 2% glutaraldehyde in 0.1 M PBS at 4°C overnight, washed with 5% sucrose in 0.1 M PBS for 15 minutes, and fixed in 1% osmium tetroxide in 0.1 M PBS for 60 minutes. Fixed embryos were washed with 5% sucrose in 0.1 M PBS for 15 minutes and dehydrated in a graded series of ethanol. The embryos were critical-point dried using liquid CO_2_, mounted on a sample holder, covered with gold, and viewed using a JSM-5800LV scanning electron microscope (JEOL).

### Enhancer analysis in zebrafish

A BAC containing the *gcm2* locus in zebrafish was identified by the *gcm2* cDNA sequence using the BAC sequence from the Sanger Institute (http://www.sanger.ac.uk/cgi-bin/blast/submitblast/d_rerio). The BAC clone 160 kb BAC (CH211-203G10), which was purchased from BACPAC Resources Center, CA, USA, had a ∼160 kb insert, of which ∼120 kb was upstream of *gcm2*. To generate *gcm2* reporter constructs, we followed the method of Lee et al. (2001) [47] using the BAC homologous recombination system (a gift from N. Copeland, National Cancer Institute, Frederick, MD, USA). To make a plasmid construct of a targeting DNA template, we subcloned Venus [48], the SV40 polyA signal, and a kanamycin resistance gene (Km^r^) in this order into pBluescript-SK. The *gcm2*-derived sequences were: 5′ arm, 5′-CTTCAGCTGAGACCAGCCATCTGTGACAAAGCGCGACAGAAACAGCAG-3′, which is located 300 bp downstream of the putative translation start site of *gcm2*, and 3′ arm, 5′-CTTTGTCCGAACTGTAACTCAGCTCTTGAATTGATTCCGTGCCGAGGTCAC-3′, which is located 354 bp downstream of the putative translation start site.

The *gcm2* locus around sequence fragments in the zebrafish genome was subcloned with the homologous recombination system [47] or amplified by PCR and cloned into the *Tol2* transposon-based vector, pTolfV, which is converted to Tol2000 [49] with the *c-fos* promoter, Venus, and the SV40 polyA signal in the opposite direction of the *Tol2* element. Each construct and the *in vitro*–synthesized capped RNA of *Tol2* [49] were co-injected as described [50]. All injected embryos at 24 hpf were observed with a GFP microscope, SteREO Lumar, V. 12 (Carl Zeiss).

### Staining with MitoTracker

MitoTracker Orange CMTMRos (Invitrogen) was used to illuminate ionocytes [51]. Zebrafish that were injected with the 160 kb BAC-Venus were stained with MitoTracker at a final concentration of 1 µM in E3 medium for 30 minutes. Observations were made with confocal microscopy using an LSM 510 microscope (Carl Zeiss).

### Injection of zebrafish embryos

Supercoiled plasmid and antisense MOs were injected into zebrafish embryos with a glass micro needle using NARISHIGE IM-30 (NARISHIGE). Control zebrafish MOs and MOs against *gcm2* were injected as described previously [24]. MOs (1 µM/embryo) were injected into cells at one- to two-cell–stage zebrafish embryos.

### Molecular cloning and probe synthesis

Total RNA was extracted from whole bodies of zebrafish (5 days old), Medaka (3–13 days old), fugu (3–7 days old), *Polypterus* (1–3 days old), sturgeon (stage 32), and *Xenopus* (stage 37) embryos. The samples then were transferred to TRIzol (Invitrogen) and treated with DNase (Invitrogen). Single-stranded cDNA was synthesized from total RNA using a PrimeScript 1st strand cDNA synthesis kit (TAKARA) according to the manufacturer's instructions.

Zebrafish cDNA clones of *gcm2* (NM_001005603), *atp1b1b* (NM_131671), *atp6v1al* (NM_201135), and *slc12a10.2* (EF591989) were isolated by PCR using the following forward and reverse primers: *gcm2*, 5′-CAGGACACGAAGCAGTATGATGCTTTTCAG-3′ and 5′-GTGAAGACATCCTTCTTTTTACCGCACTCC-3′; *atp1b1b*, 5′-CAGAGTCGCTCCTCCAGGTCTCAC-3′ and 5′-CGGAGGCTTCCCTCTTCAGTATTAC-3′; *atp6v1al*, 5′-CCAAGCTGCCTAAGATCCGAGATG-3′ and 5′-GAGTTTGTGCCGCTGCGTACCAAAG-3′; and *slc12a10.2*, 5′-CAGGGACAGCAATGTCCCTCATC-3′ and 5′-CAGATGGTGGACGATGTGAATGACG-3′. The sequence of Medaka *gcm2* was identified with the expressed sequence tag database available at the Graduate School of Science, The University of Tokyo, Tokyo, Japan, and cDNA was isolated by PCR using the following forward and reverse primers: 5′-GAGGGCGAGGAAACGGACTGCGTG-3′ and 5′-CGTTTGGGCGTGAATGGAGGCGAG-3′. The sequence of fugu *gcm2* (AB615439) was identified from the genome database available at the International Fugu Genome Consortium (http://www.fugu-sg.org/project/info.html). The cDNA was isolated by PCR using the forward and reverse primers, 5′-GCAGGACATGAAGCAGTTCGACTCG-3′ and 5′-GCTGTGTGTCCGTCGGTAGCTCACC-3′, and 3′ RACE using the SMART RACE cDNA amplification kit (Clontech). The *Polypterus* cDNA clone of *gcm2* (AB615440) was isolated by degenerate PCR using the following forward and reverse primers: 5′-GARTGGMCNGAYGGNTAYGT-3′ and 5′-ACYTTNGTNGTNGTNGTDAT-3′. The sturgeon cDNA clones for *gcm2* were amplified or isolated by degenerate PCR using the following forward and reverse primers: 5′-GARTGGMCNGAYGGNTAYGT-3′ and 5′-TCRTGNACNCCYTTNGCYTG-3′. The *Xenopus* cDNA clone of *gcm2* (AB175676) was isolated as described previously [24]. The *Polypterus* cDNA clone of *atp1b1* (AB615441) was identified from a *Polypterus* EST project (M. Okabe. unpublished results) and isolated by PCR using the following forward and reverse primers: 5′-CGAAAATGAGGGAGGATGGAAGAAG-3′ and 5′-CACATTAGGCTGTACTTTGTCCTG-3′. The sturgeon cDNA clone of *atp1b1* was isolated by degenerate PCR using the following forward and reverse primers: 5′-CARGAYMGWGTYGCWCCTCCAGGW-3′ and 5′-TTTKCCRTARTAKGGRTARTA-3′.

The PCR clone in the pGEM-T-Easy Vector (Promega) was used as a template, and a digoxigenin (DIG)-labeled antisense RNA probe was synthesized by *in vitro* transcription with T7 RNA polymerase (Promega) and DIG RNA labeling mix (Roche).

### *In situ* hybridization combined with immunohistochemistry

All samples for whole-mount *in situ* hybridization in combination with immunohistochemistry were fixed in 4% paraformaldehyde in 0.1 M PBS at 4°C overnight and were breached with breaching solution (1% H_2_O_2_, 5% formamide, 0.5× SSC∶75 mM NaCl, and 7.5 mM sodium citrate, pH 7). Fixed samples were incubated in hybridization buffer (50% formamide, 5× SSC, 500 µg/ml yeast tRNA, 50 µg/ml heparin, and 0.1% Tween-20, pH 6.0) containing DIG-labeled antisense RNA probe at 60°C overnight. The hybridized embryos were washed several times for 3 hours each with 2× SSC and 0.2× SSC and then for 10 minutes with MABT (100 mM maleic acid, 150 mM NaCl, and 0.1% Tween 20, pH 7.5). After incubation for 1 hour with blocking solution (2% Blocking Reagent (Roche) in MABT), embryos were immunoreacted overnight at room temperature with an anti-DIG Fab fragment conjugated to alkaline phosphate (Roche, 1∶5000) and rabbit anti-GFP (MBL, 1∶500). After several washes for 6 hours with MABT and three washes with NTMT (100 mM NaCl, 50 mM MgCl_2_, 100 mM Tris-HCl (pH 9.5), and 0.1% Tween-20), samples were treated with BM purple AP Substrate precipitating solution (Roche). When satisfactory coloration was achieved, samples were washed with PBST and then reacted with Alexa Fluor 488–conjugated goat anti–rabbit IgG (Invitrogen, 1∶500 in PBST) overnight at room temperature in the dark. Finally, samples were washed with PBST and photographed.

### Phylogenic analysis of the conserved sequences

The VISTA program (http://www-gsd.lbl.gov/vista/index.shtml/) was used for homology comparison. Draft genome sequence of zebrafish, *Xenopus*, chick, mouse, and human were obtained from the databases of UCSC Genome Bioinformatics (http://genome.ucsc.edu/) and the Wellcome Trust Sanger Institute, Genome Research Limited (http://www.sanger.ac.uk/).

### *In vivo* deletion assay of *gcm2* enhancer regions

A deletion assay for upstream region between −8249 and −8004 bp of *gcm2* were carried out using Gene Tailor™ Site-Directed Mutagenesis System (Invitrogen). All the fragments were cloned into pTolfV and injected into zebrafish eggs. All injected embryos at 24 hpf were observed under a GFP microscope, SteREO Lumar, V. 12 (Carl Zeiss).

## Supporting Information

Figure S1 **The sequence of** −**8 kb enhancer regions.** The possible transcription factor binding sites are shown in the boxes. The sequence was analyzed in search of transcription factor binding sites using TFSEARCH Data Base (http://www.cbrc.jp/research/db/TFSEARCHJ.html). A candidate for FOX protein binding site was previously described [52]. GATA: GATA-binding factor, SRY: sex-determining region Y gene product, FOX: Forkhead box protein, C/EBPb: CCAAT/enhancer binding protein beta, HNF-3b: hepatic nuclear factor 3beta, OCT1: octamer-binding factor 1.
(TIF)

Table S1 **Transient transgenic analysis of the** ***gcm2*** **enhancer regions.** Fragments are upstream (−) or downstream (+) of the *gcm2* loci. Each fragment was cloned into the *Tol2* transposon-based Venus expression vector (pTolfV) and injected into zebrafish eggs.
(TIF)

Table S2 ***In vivo*** **deletion assay of** −**8 kb enhancer regions.** Deletion sites, a-f, are shown in Figure S1. The sites were deleted and all the fragments were cloned into pTolfV. All reporter constructs displayed Venus positivity, indicating that the possible transcription factor binding sites of −8 kb enhancer regions in the analysis do not affect the enhancer activity.
(TIF)
