# Supplementary figures and images for: Acquisition of glial cells missing 2 Enhancers Contributes to a Diversity of Ionocytes in Zebrafish
Source: PLoS One. 2011 Aug 17;6(8):e23746. doi: 10.1371/journal.pone.0023746 (PMC3157436; doi:10.1371/journal.pone.0023746)

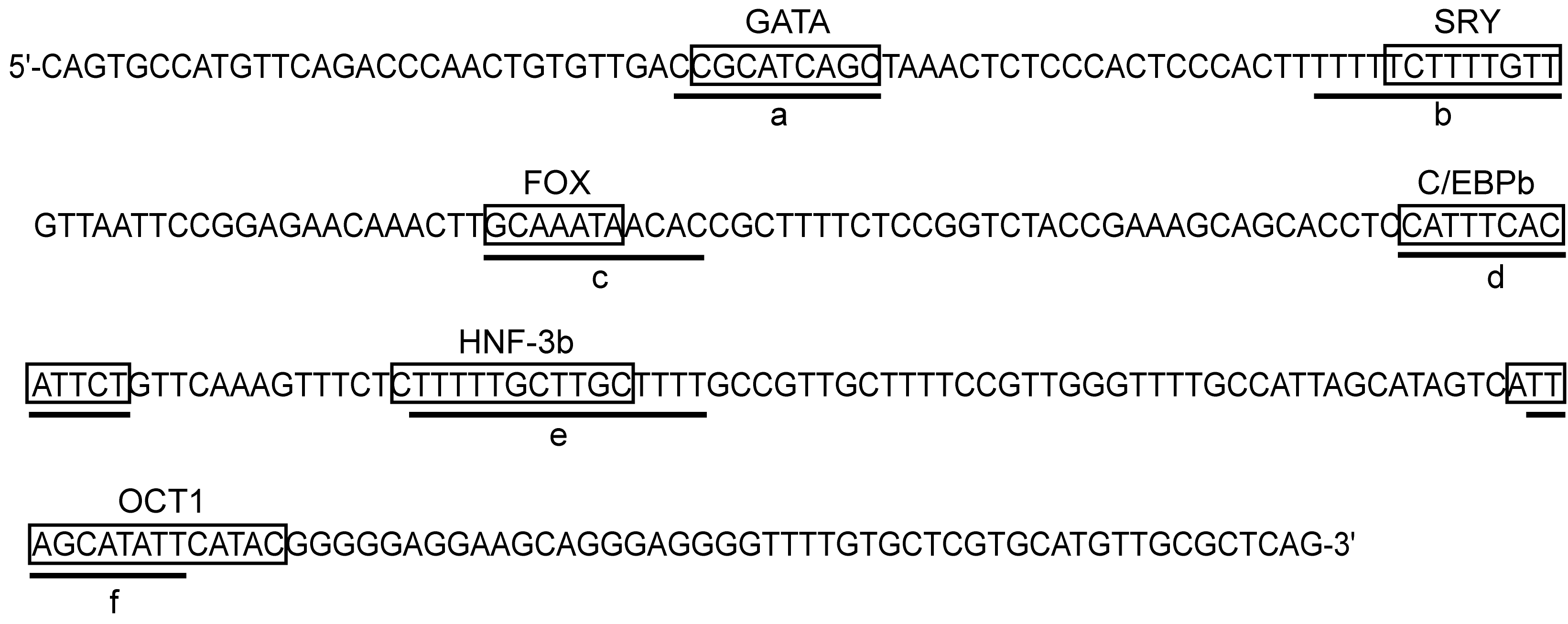

Supplement: Figure S1 — The sequence of −8 kb enhancer regions. The possible transcription factor binding sites are shown in the boxes. The sequence was analyzed in search of transcription factor binding sites using TFSEARCH Data Base (http://www.cbrc.jp/research/db/TFSEARCHJ.html). A candidate for FOX protein binding site was previously described [52]. GATA: GATA-binding factor, SRY: sex-determining region Y gene product, FOX: Forkhead box protein, C/EBPb: CCAAT/enhancer binding protein beta, HNF-3b: hepatic nuclear factor 3beta, OCT1: octamer-binding factor 1. (TIF) [file pone.0023746.s001.tif]

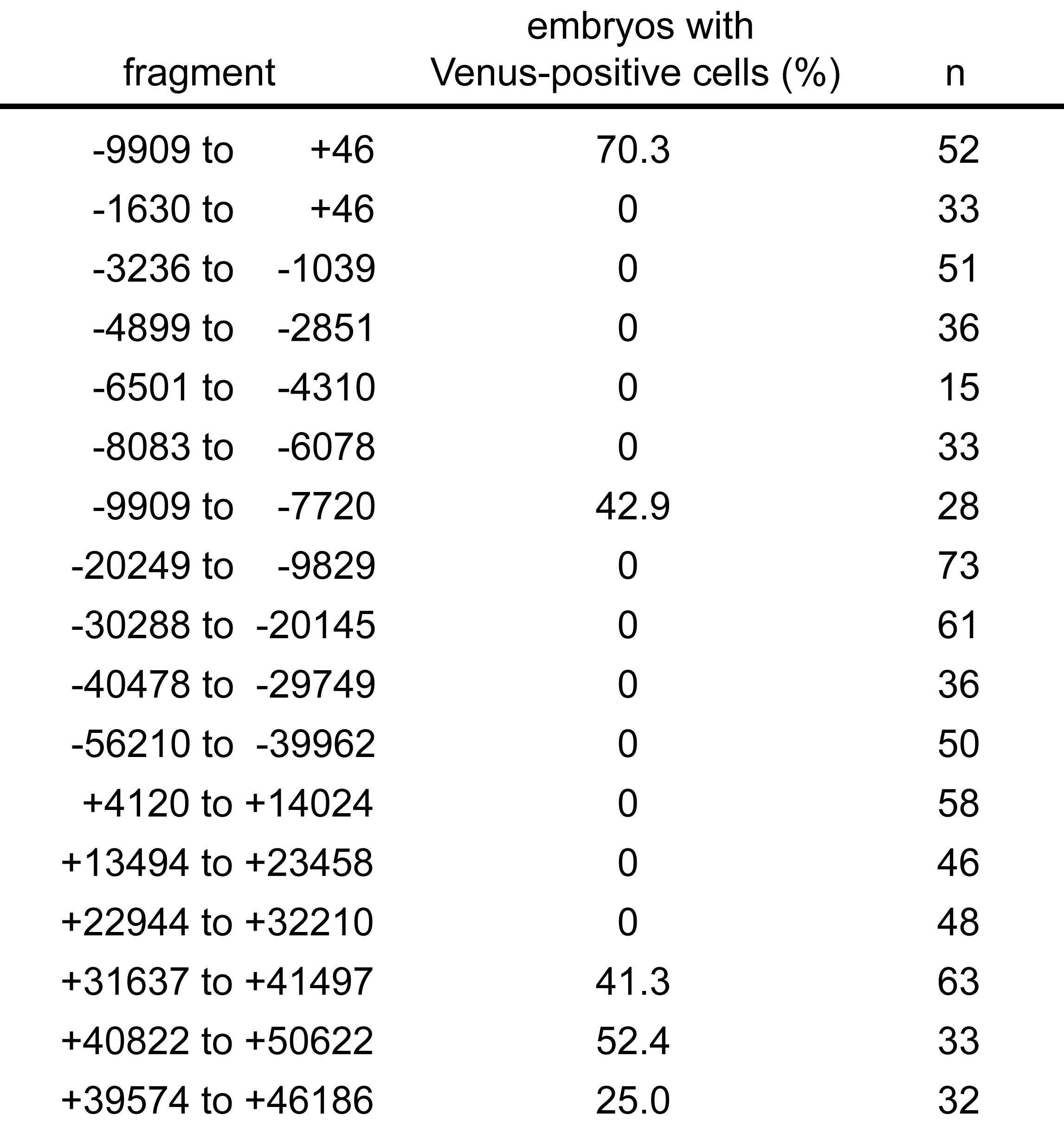

Supplement: Table S1 — Transient transgenic analysis of the gcm2 enhancer regions. Fragments are upstream (−) or downstream (+) of the gcm2 loci. Each fragment was cloned into the Tol2 transposon-based Venus expression vector (pTolfV) and injected into zebrafish eggs. (TIF) [file pone.0023746.s002.tif]

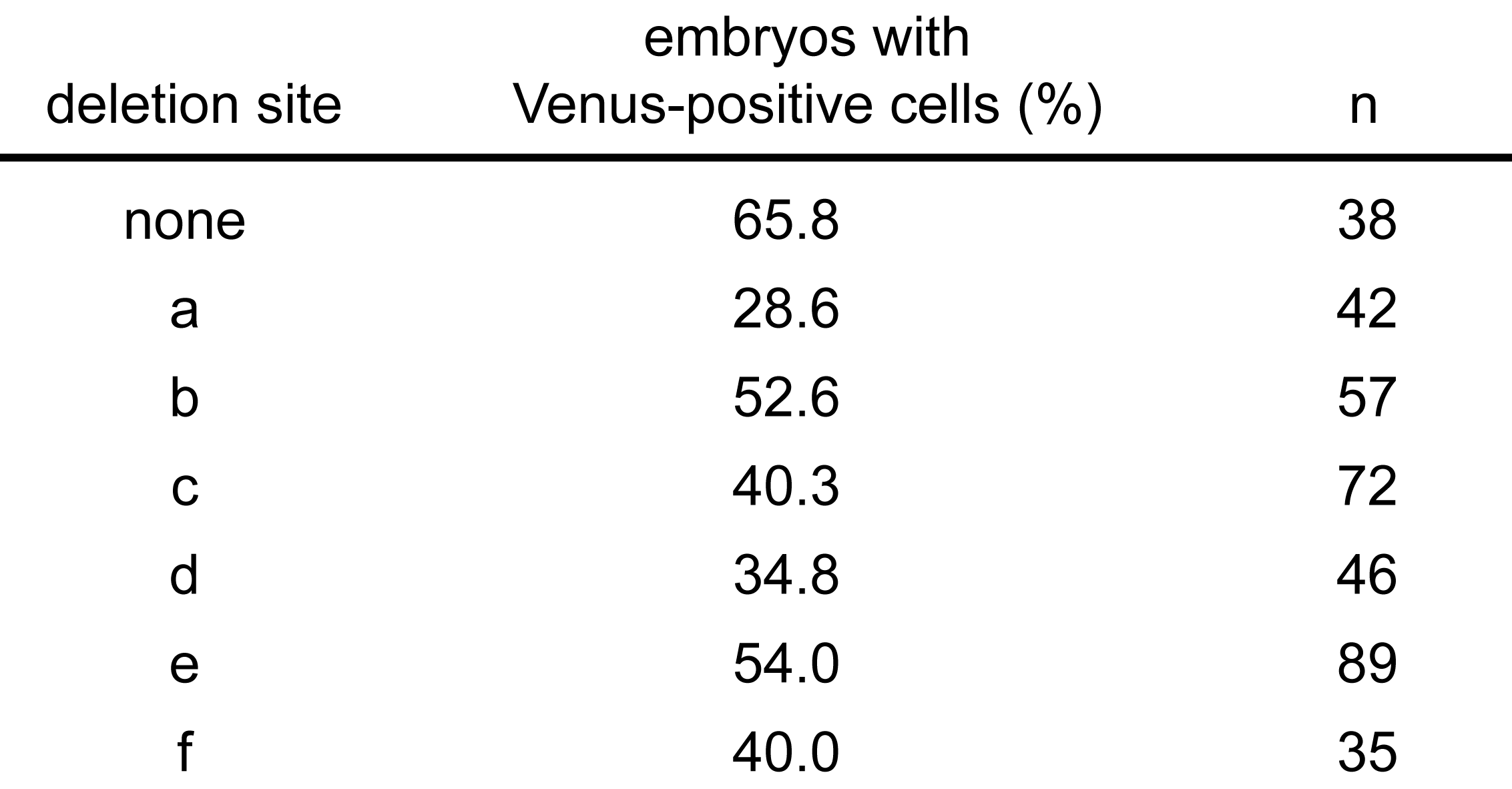

Supplement: Table S2 — In vivo deletion assay of −8 kb enhancer regions. Deletion sites, a-f, are shown in Figure S1. The sites were deleted and all the fragments were cloned into pTolfV. All reporter constructs displayed Venus positivity, indicating that the possible transcription factor binding sites of −8 kb enhancer regions in the analysis do not affect the enhancer activity. (TIF) [file pone.0023746.s003.tif]
